# Effect of Temperature on CO_2_ Adsorption onto Amine-Functionalized KIT-6 Adsorbents

**DOI:** 10.3390/molecules29133172

**Published:** 2024-07-03

**Authors:** Mariana Suba, Orsina Verdeș, Silvana Borcănescu, Alexandru Popa

**Affiliations:** Coriolan Drăgulescu Institute of Chemistry, Mihai Viteazul No.24, 300223 Timișoara, Romania; riasuba@yahoo.com (M.S.); orsinaverdes@yahoo.com (O.V.); silvana.borcanescu@gmail.com (S.B.)

**Keywords:** molecular sieves, KIT-6, 3-aminopropyltriethoxysilane, CO_2_, adsorption

## Abstract

The mesoporous silica KIT-6 was synthesized and functionalized with 3-aminopropyltriethoxysilane (APTES) by grafting at 110 °C. The composites were prepared with three different concentrations of APTES: 20, 30 and 40 wt.%. The as-prepared samples were characterized by thermal gravimetric analysis in air and nitrogen atmosphere (TG/DTA), Fourier transform infrared spectroscopy (FT-IR), X-ray diffraction and nitrogen adsorption–desorption. In this study, CO_2_ adsorption–desorption was investigated using temperature programmed desorption mass spectrometry (TPD-MS) at different temperatures. The adsorption capacity of the prepared composites is 2.23 mmol CO_2_/g at 40 °C and decreases to 0.95 mmol/g at 70 °C. Regarding the efficiency of the amino groups, the best result was obtained for APTES-grafted KIT-6 at 40 °C, with 0.512 mmol CO_2_/mmol NH_2_. The results showed good cyclical stability in adsorption capacities even after nine adsorption–desorption cycles.

## 1. Introduction

The main gases responsible for the phenomenon known as the greenhouse effect are steam, carbon dioxide, methane and ozone. Carbon dioxide amounts to about 79% of the total and its concentration is increasing dramatically because of human activities, for instance: massive deforestation, coal burning, natural gas, petroleum fuels and the industrialization of most economic sectors. In order to reduce and mitigate the negative effects of CO_2_, research has focused on obtaining materials with adsorption–desorption properties to capture and store CO_2_ through different technologies [1].

The chemical properties of various oxides (of alkaline, transition and alkaline earth metals) have led to extensive research of their use in the adsorption–desorption of CO_2_. Their thermodynamic properties are used to calculate the thermodynamic equilibrium of the CO_2_ absorption–desorption cycle according to the values of the heat of reaction and chemical potential; however, this is not the only criterion for validating a material, as other factors must also be taken into account such as cost, regeneration temperature, durability, etc. [2,3,4]. Although metal oxides have good interactions with CO_2_, there are also a number of disadvantages to their use, such as quite difficult absorbent regeneration, the use of high temperatures and reduced durability in some cases. Utilizing these important selection criteria put forward adsorbents based on MgO, showing that they have fast adsorption speed, stability and good reversibility of regeneration during 10 cycles without decreasing work capacity, but they also a significant influence regarding the content of carbonate and nitrate salts [5].

Mesoporous and microporous materials based on SiO_2_ are the most often used in CO_2_ adsorption–desorption studies. The adsorption of CO_2_ is higher in the case of microporous materials, but mesoporous ones are preferred because they can be used as a support which can be functionalized by different amines. Amino groups favor CO_2_ adsorption due to the physical and chemical interactions between functionalized species and CO_2_ molecules [6,7,8,9].

In the last three decades, the synthesis of mesoporous materials has gained considerable momentum and the main morphologies in this family are: MCM-41 [10,11] with hexagonal symmetry P6mm and mesochannels that are interconnected by micropores, MCM-48 [12] with Ia3d cubic symmetry and MCM-50 [13], which is characterized by a lamellar structure.

One of the most studied mesoporous materials is SBA-15, which has a hexagonal structure and P6mm symmetry with thicker walls compared to the other mesoporous materials, leading to the ability to have clearly superior mechanical and hydrothermal resistances due to the large pores. Thus, SBA-15 mesoporous sieves are among the most widely studied as a catalytic support for active phase dispersion [14,15,16]. Mesoporous silica of the KIT-6 type, characterized by a three-dimensional cubic Ia3d structure with large interconnected pores and large specific surfaces, is also being studied more and more intensively [8,17]. KIT-6 silica has a great advantage given by the silanols groups that can be functionalized post-synthesis, leading to modification of the surface properties to favor the adsorption–desorption of gases.

Silica materials functionalized with amines, named aminosilica, are some of the most important and widely studied materials with adsorbent properties used for CO_2_ capture [18]. Amine functionalization of silica can be performed by the impregnation method, when the amines are physically load onto the silica support, or by the grafting method, through covalent bonding. At the equlibrium point, amine-impregnated silica presents higher CO2 uptake because of its higher amine loading compared with amine-grafted samples. Therefore, amine-impregnated silicas may have slower adsorption kinetics and lower thermal and hydrothermal stability, leading to amine evaporation.

The most used adsorbents in post-combustion capture (PCC) of CO_2_ are based on amines, like monoethanolamine (MEA), diethanolamine (DEA), 3-aminopropyl triethoxysilane (APTES), tetraethylenepentamine (TPEA), polyethylenimine (PEI), etc. [19,20,21,22]. Using these amines for high CO_2_ capture efficiency results in some disadvantages regarding the high levels of equipment corrosion and the high energy consumption needed for amine regeneration. All of these requirements represent additional costs for the application of this method [23].

The functionalization of mesoporous sieves with amines with different number of nitrogen atoms led to obtaining materials with varying CO_2_ adsorption capacities. Vilarrasa-Garcia E et al. [24] studied MCF-type porous silica by grafting with 3-aminopropyl triethoxysilane (APTES) and by impregnation with polymers rich in amine groups PEI or TEPA, and found that grafting with APTES improves CO_2_ adsorption and physisorption has a main role compared to chemosorption. The increase in temperature had a negative effect for MCFs grafted with APTES, while in the case of samples impregnated with PEI and TEPA, the adsorption was assigned to chemical interactions between the amine species and carbon dioxide. In the case of MCF materials impregnated with PEI or TEPA, the chemisorption process controlled the CO_2_ retention over the physisorption process and as a result, CO_2_ uptake was enhanced by increasing the temperature. The authors concluded that the CO_2_/N molar ratio was considerably lower for the PEI and TEPA grafted samples compared to the APTES-grafted samples because of the lesser availability of the amino groups. The stability of the investigated materials was reflected the nine CO_2_ adsorption–desorption cycles.

Runa Dey et al. [14] studied adsorption–desorption kinetics and the stability of a mesoporous SBA-15 material impregnated with polyethylenimine (PEI), obtaining an optimal amine loading of 50% with a CO_2_ adsorption capacity between 2.79 and 3.09 mmol∙g^−1^. In order to improve CO_2_ adsorption by adding moisture to the atmosphere, they used 10% pre-humidified CO_2_ at 75 °C. The authors concluded that the CO_2_/N molar ratio was considerably lower for the PEI- and TEPA-grafted samples compared to the APTES-grafted samples because of the lesser availability of the amino groups. The stability of the investigated materials was reflected by nine CO_2_ adsorption–desorption cycles.

The researchers in the field directed their research towards a series of mesoporous materials that were functionalized with various amines in order to increase the adsorption–desorption capacity of carbon dioxide, studying various parameters such as the reaction medium, temperature, molar ratios, the influence of pore sizes, etc. Liu Y. et al. [25] functionalized KIT-6 mesoporous silica with pentaethylenehexamine (PEHA) and the obtained results showed that the pore size, pore volume and surface area of the adsorbents decreased after loading with PEHA, while the basic structure of the KIT-6 pores remained unchanged. The amount of adsorbed carbon dioxide increased with increasing temperature, obtaining a maximum adsorption at 70 °C, which remained almost constant even after 10 cycles of adsorption–desorption, while above this temperature the adsorption capacity decreased.

Son W.J. [26] et al. synthesized the mesoporous silicas MCM-41, MCM-48, SBA-15, SBA-16 and KIT-6 and impregnated all of them with 50% polyethyleneamine (PEI) in methanol. They studied CO_2_ adsorption–desorption in cycles of 150 min at a temperature of 75 °C and obtained reversible adsorption–desorption behavior for all tested types of mesoporous silica. The KIT-6 mesoporous silica presented the best stability in three consecutive tests over a duration of 900 min and the highest CO_2_ adsorption capacity of 135 mg/g due to its larger pores in a 3D arrangement; the order of effectiveness was: KIT-6 > SBA-16 ≈ SBA-15 > MCM-48 > MCM-41.

The influence of the reaction environment on amine-grafted adsorbents was investigated by Anyanwu et al. [27], who found that the presence of water had much better effects vis a vis alcohol on SBA-15 loading and on other adsorbents. A continuous increase in water concentration led to polymerization and a higher loading of amines without signs of a plateau and also showed superior stability after several cycles of adsorption–desorption.

In addition to the mesoporous silicas previously described, there are many novel nanoporous materials for CO_2_ capture which show good performance, such as porous carbon materials, heteroatom-doped biomass carbon materials, MOFs, COFs and so on. Rehman et al. [28] made a series of heteroatom-enriched porous carbons using KOH as a porogen and activating agent, while urea/thiourea was used as an N/N and S dopant. A cellulose-based highly porous carbon framework (1026 m^2^/g, 0.7135 cm^3^/g) with a narrow micropore stucture (<0.94 nm) significantly contributed to an efficient CO_2_ uptake of ~297.1 mg/g at 0 °C and 193.7 mg/g at 25 °C/1 bar. An excellent CO_2_/N_2_ selectivity was obtained for Cell-TK at ~110 at 25 °C/1 bar. These results were achieved due to the large number of narrow micropores with high adsorption potential, together with high N and S contents, which produced a better affinity for CO_2_ molecules through Lewis acid–base interactions. Sani et al. [29] presented recent progress in the development of different types of covalent organic frameworks (COFs), which are a class of crystalline porous materials built entirely from light elements (C, H, O and N), as well as their application as catalysts for different types of CO_2_ fixation reactions. These thermally stable COFs with excellent surface areas and volumes are now used for the conversion of CO_2_ to CO by photochemical or electrochemical reduction.

Muchan et al. [18] studied CO_2_ adsorption–desorption on mesoporous MCM-41, SBA-15 and KIT-6 silicas functionalized with APTES. The adsorption was carried out under conditions of normal temperature and atmospheric pressure using 15% CO_2_, and desorption was carried out at 100 °C under N_2_ balance. The three silicas modified with APTES presented an improvement in adsorption capacity, demonstrating that the primary amine present in the structure can carry out a rapid chemical reaction with CO_2_ in the mesopore channels, but the results diminished in the middle and final stages due to CO_2_ transport through smaller pore channels. KIT-6 can be considered a promissing adsorbent material due to its large pore size and large pore volume. Investigations show that KIT-6 presents a reasonable adsorption capacity with a high adsorption rate. In addition, it can also be regenerated with an efficiency of 99.72% using a 12.07 kJ/mmol CO_2_ heat load for regeneration.

In this work, mesoporous cubic Ia3d KIT-6 was prepared and functionalized with 3-aminopropyltriethoxysilane (APTES) by the grafting method. The composites were prepared with three different concentrations of APTES: 20, 30 and 40 wt.%. The obtained materials were studied by thermogravimetric analysis (TG/DTA), FT-IR spectroscopy and X-ray diffraction at low angles, and surface areas were determined by the BET method. The adsorption capacity (mg CO_2_/g ads) and the efficiency of amino groups (mol CO_2_/mol NH_2_) were measured using a thermo analyzer system coupled with mass spectrometry (MS) in the temperature range of 40–70 °C.

In our work, we performed a detailed study of the influence of temperature and functionalization agent concentration on the adsorption capacity (mg CO_2_/g ads) and the efficiency of amino groups (mol CO_2_/mol NH_2_) for mesoporous silica KIT-6 composites. We combined two methods, thermal analysis and mass spectrometry, for the investigation of CO_2_ adsorption–desorption. It is important to underline that the change in the amount of adsorbed and desorbed CO_2_ was determined from the MS spectra for all stages of the CO_2_ adsorption–desorption process. The stability and recyclability of the studied amino-functionalized materials were also tested for a longer period of time, in order to demonstrate their usability for practical applications.

## 2. Results and Discussion

**Figure 1:**
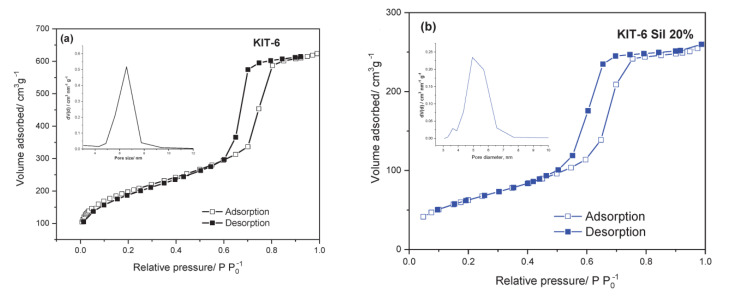

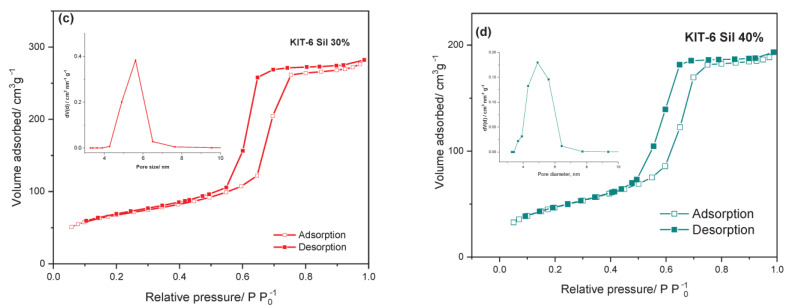
Nitrogen adsorption–desorption of parent KIT-6 (**a**), KIT-6 Sil 20% (**b**), KIT-6 Sil 30% (**c**) and KIT-6 Sil 40% (**d**).

The textural parameters of the parent and amino-functionalized materials are presented in Table 1. The results show that the surface area and the other textural parameters of KIT-6 decreased after adding APTES in three different concentrations: 20, 30 and 40 wt.%. The modified KIT-6 samples are denoted as KIT-6 Sil. The reduction in surface area may be due to the intensification of the silica particles’ agglomeration and/or the filling of pores after the addition of APTES. All composites show a type IV isotherm with H1 hysteresis and a sharp increase in volume adsorbed at p/p_0_ = 0.5–0.9, characteristic of highly ordered mesoporous materials (Figure 1a–d). The micropore volume and micropore area parameters were calculated by the V-t method, and the results for KIT-6 were as follows: V_mp_ = 0.07 cm^3^ and A_mp_ = 147.9 m^2^/g, respectively (Table 1). After functionalization with APTES, for the KIT-6 Sil 20–40% samples, the values of the textural parameters of the micropores decreased, probably as a consequence of the micropores filling or the agglomeration of silica particles. 

**Figure 2:**
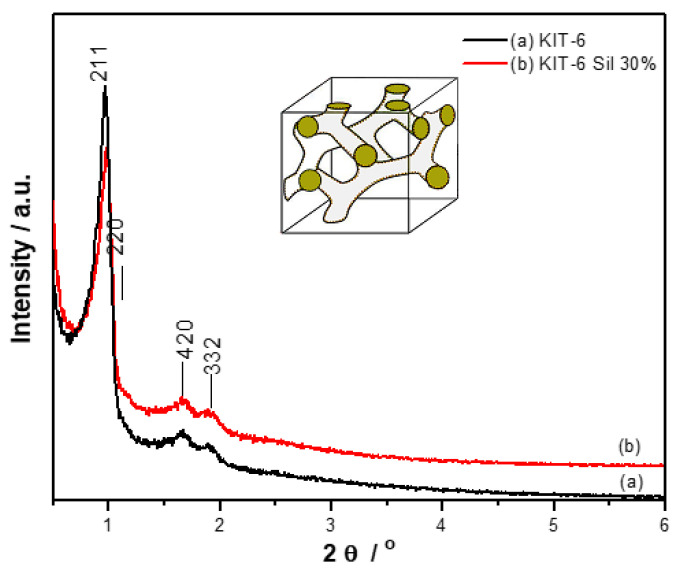
XRD spectra of KIT-6 (a) and KIT-6 Sil 30% (b).

Figure 2 shows the diffractograms for KIT-6 (a) and KIT-6 Sil 30% (b) obtained at low angles in the range 2θ = 0–6°. The presence of four diffraction peaks at 0.96°, 1.16°, 1.69° and 1.92° corresponds to the Miller indices (211), (220), (420) and (332), confirming the *Ia3d* cubical mesoporous structure specific to KIT-6 sieves [30,31]. With the introduction of APTES, a slight decrease in the tipping points was observed, which suggests that the grafting operation was successful.

**Figure 3:**
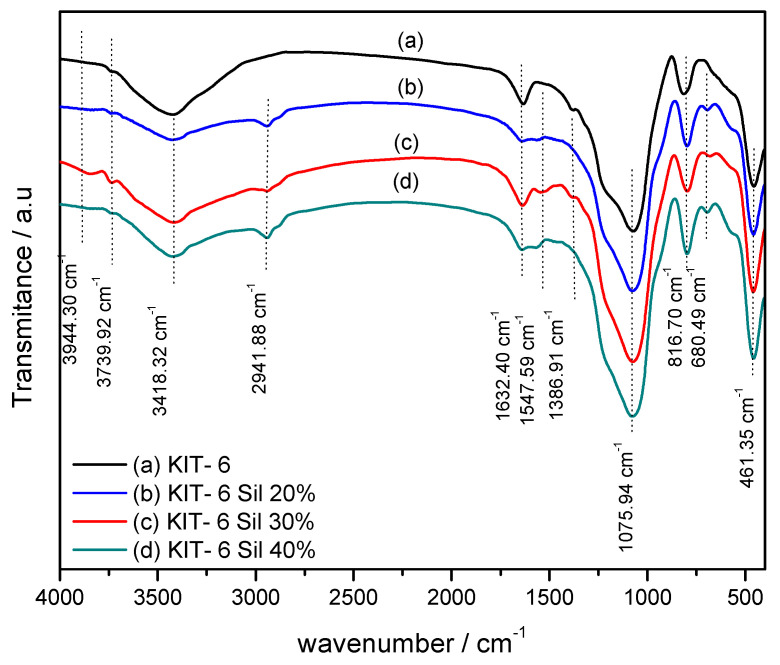
The FTIR spectra of KIT-6 (a), KIT-6 Sil 20% (b), KIT-6 Sil 30% (c) and KIT-6 Sil 40% (d).

As is illustrated in Figure 3, the vibration bands of the parent KIT-6 and KIT-6 Sil 20–40% were determined by Fourier-transform infrared analysis. Generally, the IR absorption bands correspond to the stretching frequencies of the inorganic functional groups in all samples. It can be seen that all samples have absorption bands around 3739, 3418, 1632, 1386, 1075, 816 and 461 cm^−1^.

The band at 1632 cm^−1^ is due to water deformation modes and the respective OH stretching modes are responsible for the band at 3418 cm^−1^. A contribution to the latter band also comes from the OH of some silanols interacting with adsorbed H_2_O, leading to a red shift of their OH modes. The band at 3739 cm^−1^ is typical on internal silanols [31,32]. The bands that can be observed at 1075 cm^−1^ and the shoulder present at 1386 cm^−1^ on the FT-IR spectra can be atributed to asymmetric stretches of the Si–O–Si bonds. The smaller peak observed at 816 cm^−1^ coresponds to symmetrical stretches of the Si–O–Si bonds. The band present at 461 cm^−1^ refer to the symmetrical and asymmetric stretches of the Si–O bonds of the Si–OH groups [32].

The FT-IR spectra of KIT-6 Sil 20–40% (Figure 3b–d) show the presence of peaks around 680 cm^−1^ and 1547 cm^−1^, corresponding to –NH- and –NH_2_ bending vibrations that are absent in the parent KIT-6. Also, N-H stretching modes are expected around 3200 cm^−1^ and contribute to the band at 3418 cm^−1^. The adsorption band at 2941 cm^−1^ corresponds to the asymmetric deformation of the -CH_2_ groups present in the propyl chain of APTES. The FT-IR results indicate the successful loading and grafting of APTES onto the surface.

**Figure 4:**
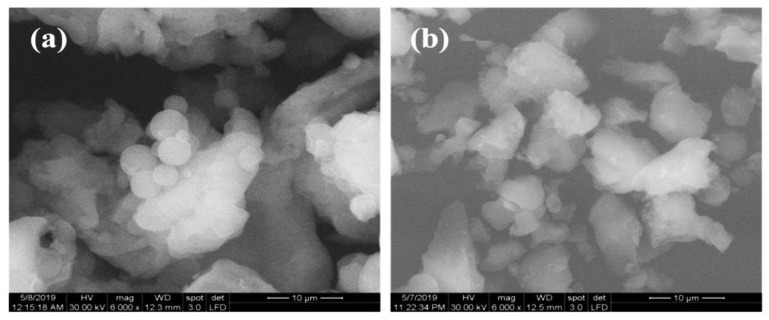
SEM micrographs of KIT-6 (**a**) and KIT-6 Sil 30% (**b**).

Scanning electron micrograph (SEM) images were used to analyze the morphology of the KIT-6 and KIT-6 Sil 30% particles (Figure 4). The picture of KIT-6 (Figure 4a) shows an agglomeration of spherical particles characteristic of an orderly network with an Ia3d structure type [14,33]. It can be seen that the spherical morphology is modified by the adding of APTES, with the spheres becoming rougher and more dispersed, which indicates that the grafting has taken place successfully (Figure 4b).

### 2.1. Thermal Analysis

**Figure 5:**
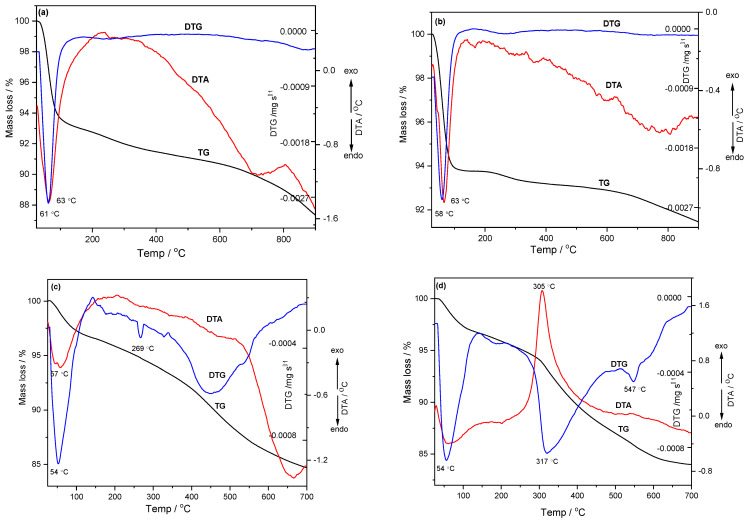
TG–DTG–DTA curves for (**a**) KIT-6 (nitrogen), (**b**) KIT-6 (air), (**c**) KIT-6 Sil 30% (nitrogen) and (**d**) KIT-6 Sil 30% (air).

The thermal stability was investigated by TG-DTA-DTG methods in the temperature range of 25–900 °C in the case of the KIT-6 mesoporous sieve, and the mesoporous sieves functionalized with 3-aminopropyl triethoxysilane (APTES) were tested in the temperature range of 25–700 °C. For the KIT-6 and KIT-6 Sil 20–40% mesoporous sieves, the thermal stability studies were performed in an inert atmosphere (nitrogen), as well as in an oxidizing atmosphere (air), with a heating rate of 10 °C /min. From the thermal analysis of KIT-6 in the oxidative as well as inert atmospheres, three stages of mass loss could be found (Figure 5a,b). In the first stage, the mass loss was due to the elimination of physically adsorbed water and residual solvents from the synthesis process, a fact supported by the endothermal effect at 63 °C. In the second stage, a significant mass loss was observed, which was due to the degradation of the organic matrix and the elimination of chemosorbed water, while in the third stage, the mass loss corresponded to the condensation of silanol groups remaining on the surface and in the material pores [34].

From the thermal analysis of KIT-6 Sil 30% in the inert and oxidizing atmosphere, the endothermic effects at 54 °C (Figure 5c,d) corresponded to the elimination of physically adsorbed water and residual solvents. At temperatures above 200 °C, it can be observed that the exothermic effect at 305 °C in the oxidizing atmosphere (Figure 5d) was much stronger than the one obtained in the inert atmosphere (Figure 5c), which suggests that the oxidation and decomposition of the APTES amination agent occurred faster, with a faster mass loss and a noticeably lower thermal stability.

These results demonstrate the relatively good thermal stability of the investigated KIT-6 and KIT-6 Sil materials. In agreement with these results, TPD measurements regarding the adsorption–desorption process of CO_2_ were performed in a narrower temperature range, between 40 and 70 °C.

The mass loss between temperatures 250 and 700 °C in the case of the KIT-6 Sil 30% sieve was significantly higher (11% in N_2_ and 13% in air) than in the case of the parent KIT-6, which suggests that the functionalization of the KIT-6 sieve was successfully achieved.

### 2.2. The Adsorption–Desorption Process of CO_2_

**Figure 6:**
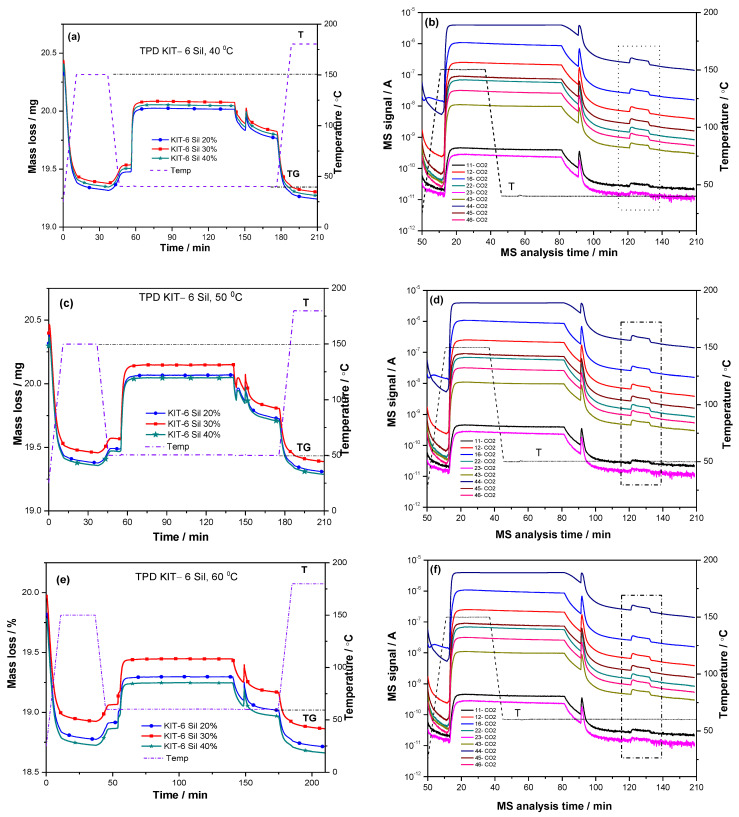

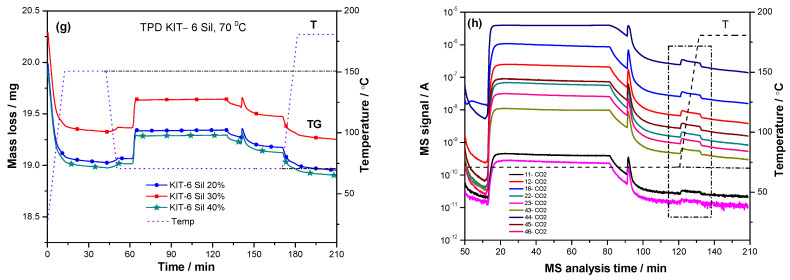
CO_2_ adsorption–desorption steps of functionalized samples KIT-6 Sil 20–40% with an adsorption isotherm at 40 °C (**a**), 50 °C (**c**), 60 °C (**e**) and 70 °C (**g**) and MS for KIT-6 Sil 30% at 40 °C (**b**), 50 °C (**d**), 60 °C (**f**) and 70 °C (**h**).

The adsorption–desorption process of CO_2_ was studied for amine-grafted KIT-6 Sil 20–40% using the TPD program. Since the non-amine-functionalized KIT-6 mesoporous sieve did not show a carbon dioxide capture activity, the CO_2_ adsorption–desorption process was studied for amine-grafted KIT-6 Sil 20–40% at different temperatures (Figure 6a–f) by thermogravimetric analysis. The mass gain of the samples in mmol of CO_2_ per gram of adsorbent during the adsorption process represents the carbon dioxide adsorption capacity. The steps of the adsorption–desorption processes at temperatures of 40, 50, 60 and 70 °C for KIT-6 Sil 20–40% are the following: first, each sample was pretreated in a stream of N_2_ at 150 °C for 30 min to remove physically adsorbed water, as well as possible impurities, then, the temperature was reduced to the desired adsorption temperature and maintained under the same nitrogen flow. The next step was the exposure of the samples to the adsorption gas mixture, 30% CO_2_/N_2_ (70 mL/min), which was maintained for 1.5 h. The last step after the completion of the adsorption process was the maintenance of the samples for 30 min in N_2_ for the removal of the physically adsorbed CO_2_.

After cleaning the surface of physically adsorbed CO_2,_ the next stage was the desorption of chemisorbed CO_2_ from the amine-grafted samples of KIT-6 Sil 20–40%. This step was carried out from the adsorption temperature up to 180 °C, with a temperature increase of 10 °C/min and with an isotherm at 180 °C for 30 min. The change in the amount of desorbed CO_2_ was determined (at certain mass-to-charge ratios, m/z = 46, 45, 44, 43, 23, 22, 16, 12 and 11) from the MS spectra (Figure 6b,d,f,h) for all stages of the CO_2_ adsorption–desorption process.

The signal characteristic for CO_2_ on the MS spectra increased during the adsorption process and was maintained at a constant for 90 min during the exposure process of the CO_2_/N_2_ gas mixture. When the exposure to the gas mixture was interrupted, the MS signal characteristic for CO_2_ decreased. Then, the sample was kept for 30 min under a continuous N_2_ atmosphere. In this stage of the adsorption–desorption process, the physisorbed CO_2_ was removed from the sample. Increasing the temperature up to 180 °C (the selected area in the Origin plots in Figure 6b,d,f,h), the signal characteristic for CO_2_ increased again during the desorption step of the chemisorbed CO_2_.

The amounts of CO_2_ captured on the KIT-6 Sil 20–40% adsorbents at different temperatures are shown in Figure 6a,c,e,g. It can be seen that with an increase in the temperature, the adsorption capacity and the efficiency of the amino groups decreased for all concentration of APTES: 20, 30 and 40 wt.%. In the case of KIT-6 Sil 30%, the adsorption capacity and the efficiency of the amino groups decreased from 2.23 to 0.95 mmol CO_2_/g SiO_2_ and from 0.51 to 0.22 mmol CO_2_/mmol NH_2,_ respectively. At 40 °C, an adsorption capacity of 2.23 mmol CO_2_/g SiO_2_ and an efficiency of the amino groups of 0.51 mmol CO_2_/mmol NH_2_ represent the best results for KIT-6 Sil 30%; these results are clearly superior to those at the other temperatures of 50, 60 and 70 °C, as can be seen in Figure 6a,b. The formulae used to calculate the above parameters, including adsorption capacity and the efficiency of amino groups are published elsewhere [15].

The influence of functionalization agent concentration was studied for all concentrations of APTES: 20, 30 and 40 wt.%. The adsorption capacity and the efficiency of the amino groups of the captured CO_2_ at 40 °C for all composites are summarized in Table 2. At the lowest temperature (40 °C), an adsorption capacity of 2.23 mmol CO_2_/g SiO_2_ and an efficiency of amino groups of 0.51 mmol CO_2_/mmol NH_2_ represent the best results for KIT-6 Sil 30%, which was clearly superior to the other concentrations of APTES.

From the obtained results, it can be concluded that the values for both adsorption capacity (mmol CO_2_/g adsorbent) and the efficiency of the amino groups (mmol CO_2_/mmol NH_2_) strictly depend on the investigated temperature and, to a lesser extent, on the functionalization agent concentration. At lower investigated temperatures, 40 and 50 °C, respectively, higher values of CO_2_ adsorption were obtained. By increasing the temperature investigation range to 60 and 70 °C, the values for CO_2_ adsorption decreased. The achieved results for mesoporous silica KIT-6 are comparable or even superior to results existing in the literature. Some of these results regarding the adsorption capacities obtained for different functionalized mesoporous molecular sieves using different amines (APTES, PEI and PEHA) are summarized in Table 3.

**Figure 7:**
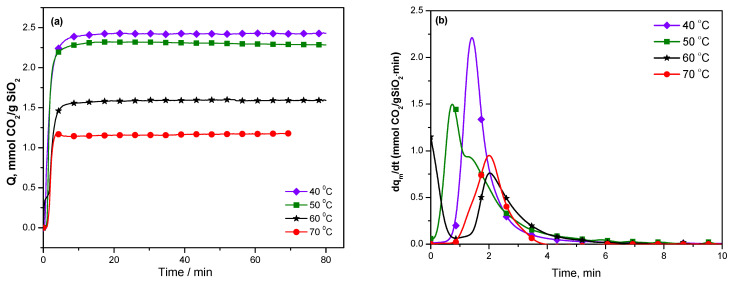
Carbon dioxide adsorption (**a**) and carbon dioxide adsorption rate of KIT-6 Sil 30% (**b**) at temperatures between 40 and 70 °C.

The variations of the CO_2_ adsorption with time and its time derivative—which is considered as a measure of adsorption rate—are shown in Figure 7 for KIT-6 Sil 30% at temperatures between 40 and 70 °C. The CO_2_ adsorption rate reached the maximum values for KIT-6 Sil 30% at 40 and 50 °C and then decreased at higher temperatures.

Álvarez et al. [39] studied the sources of activation of the CO_2_ molecule on catalytic surfaces such that electricity, light and/or temperature can be external activators. We can say that a temperature of 40 °C is the most suitable for the KIT-6 type mesoporous sieve functionalized with APTES, as it is the most thermally stable and can be used as an adsorbent for CO_2_. The type of amines used can influence the reaction mechanism for CO_2_ adsorption [40]. By incorporating some basic organic groups (in this case, APTES) into KIT-6 type mesoporous silica, surface chemistry changes occur, leading to an increase in the CO_2_ adsorption capacity.

According to the literature data [41], the interaction of amines with CO_2_ generates a reversible reaction of carbamates by the formation of 1,3-zwitterion, R-NH2^+^COO^−^, which is often characteristic under dry conditions. The reaction mechanism with the formation of 1,3-zwitterion resulting from the interaction between CO_2_ and amines can be expressed as follows:

R-NH_2_ + CO_2_ → R-NH_2_^+^COO^−^ (1)

R-NH_2_^+^COO^−^ + R-NH_2_ → R-NHCOO^−^ + RNH_3_^+^ (2)

This type of reaction requires two amine groups per one CO_2_ molecule, so the CO_2_/N ratio (amine efficiency) is found to be in accordance with the density of the amine groups.

The regeneration reaction of the KIT-6 mesoporous molecular sieve and CO_2_ desorption can be expressed by the following reaction:

R-NH-COO^−^ + R-NH_3_^+^ + Heat→ CO_2_ + 2R-NH_2_ (3)

**Figure 8:**
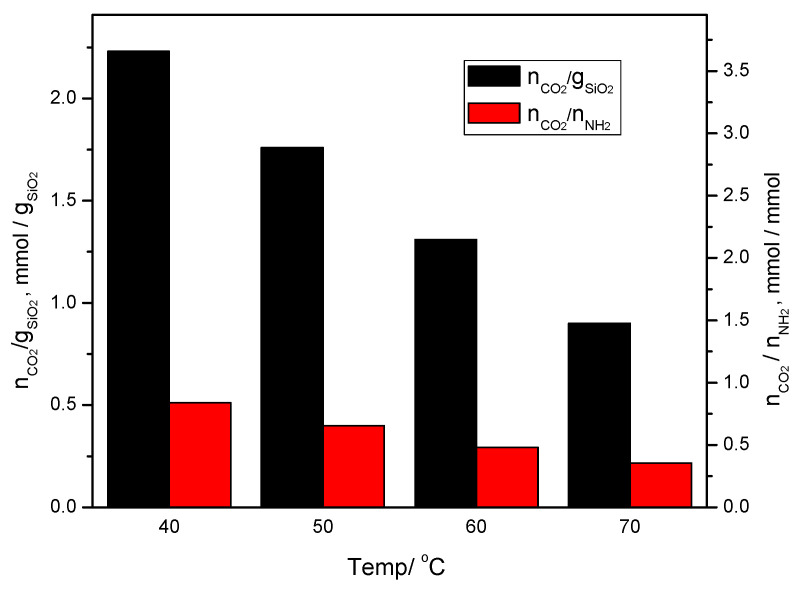
The adsorbed amounts of CO_2_ by KIT-6 Sil 30% at 40–70 °C.

From the mass loss during the desorption process measured in mmol CO_2_/mmol NH_2_, the efficiency of the adsorbent KIT-6 Sil mesoporous molecular sieve functionalized with APTES was calculated at different temperatures. As can be seen from Figure 8, the adsorption capacity (mmol CO_2_/g ads) and the amino group efficiency (mmol CO_2_/mmol NH_2_) were higher in the case of the KIT-6 Sil functionalized mesoporous molecular sieve at 40 and 50 °C.

In order to be able to say that a material has a practical utility (value), several criteria must be taken into account, such as high adsorption–desorption capacity, good selectivity and, last but not least, the greatest possible stability during prolonged operation for CO_2_ capture.

**Figure 9:**
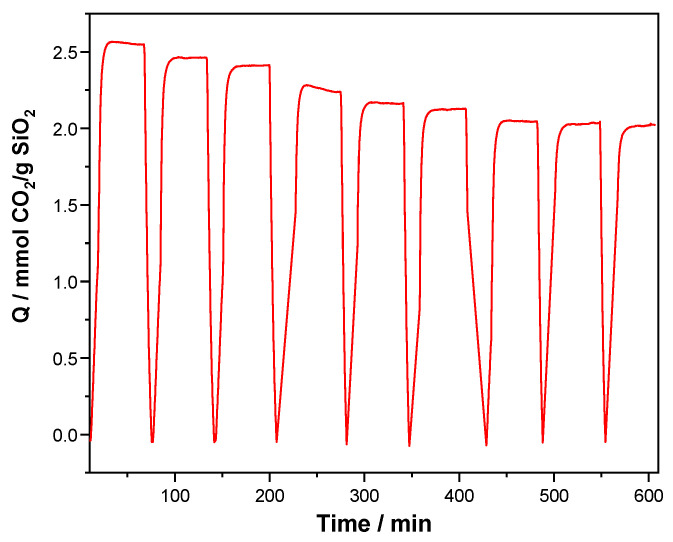
CO_2_ adsorption–desorption cycles of functionalized samples of KIT-6 Sil 30% with adsorption at 40 °C.

Figure 9 shows the behavior of KIT-6 Sil 30% with the highest CO_2_ adsorption rate at 40 °C for a duration of nine cycles. The KIT-6 Sil 30% sample was pretreated in flowing N_2_ at 120 °C. The samples were kept in this environment for 10 min, then cooled down to the adsorption temperature, 40 °C, and exposed further to 30% CO_2_ in N_2_ for 40 min. The CO_2_ desorption was achieved by heating the sample to 120 °C at a rate of 10 °C/min.

As can be seen in Figure 9, the KIT-6 Sil 30% adsorbent (40 °C) showed a high adsorption capacity and good stability over nine cycles of adsorption and desorption. After nine cycles of experimental tests, the adsorption capacities of KIT-6 Sil 30% showed only a 1.46% decrease. This decrease of 1.46% can be assigned to the regeneration conditions (120 °C), which can be responsible for removing small proportions of N-species that are not bound strongly enough to the KIT-6 type mesoporous molecular sieve surface through electrostatic interactions.

## 3. Materials and Methods

### 3.1. Sample Preparation

The KIT-6 mesoporous material was prepared by the method developed by Kleitz et al. [42]. A detailed description of the KIT-6 preparation was presented in our previous work [43]. A brief description of the KIT-6 Sil sample preparation method is described as follows: 0.5 g of KIT-6 was dispersed in 50 mL of toluene; after the complete dissolution of KIT-6, 0.79 mL of 3-aminopropyl triethoxysilane (APTES) was added drop by drop. The suspension was refluxed at 110 °C for 12 h. The resulting solid material was collected by filtration, washed with ethanol and dried at 80 °C. The grafting reaction took place at 110 °C for 5 h. After filtration and drying, the absorbents were obtained as white solids. Also, we prepared composites with three different concentration of APTES: 20, 30 and 40 wt.%, denoted as KIT-6 Sil 20%, KIT-6 Sil 30% and KIT-6 Sil 40%.

### 3.2. Characterization Methods

X-ray diffraction data were recorded with a XD 8 Advanced Bruker diffractometer (Karlsruhe, Germany) operating with CuK_α_ radiation in the range 2θ = 0.5–6°. Nitrogen adsorption–desorption isotherms were obtained at −196 °C after degassing the prepared samples at 200 °C for 6 h to remove the moisture. S_BET_ was determined from the BET method using a Quantachrome instrument, Nova 2000 series (Boynton Beach, FL, USA).

SEM images were performed using a Joel JMS 6460 LV instrument (Tokio, Japan) equipped with an Oxford Instruments EDS (energy dispersive spectroscopy) analyzer. The FT-IR absorption data were collected in the spectral range of 4000–400 cm^−1^ using a Jasco 430 spectrometer (Tokio, Japan; 256 scans at a resolution of 2 cm^−1^, KBr pellets).

TGA was recorded using a Mettler TGA/SDTA 851/LF/1100 (Columbus, OH, USA) apparatus coupled with mass spectrometry (MS). The measurements were performed in a dynamic air atmosphere, 50 mL/min, using alumina plate crucibles of 150 µL. The tests of CO_2_ adsorption–desorption using a TPD (temperature program desorption) program were obtained using the same TGA unit coupled with MS. The adsorption experiments were performed using pure CO_2_ and 30% CO_2_ in N_2_ at 1 atm., and for the desorption step N_2_ was used as the purge gas. The prepared samples were first pretreated in a N_2_ atmosphere at 150 °C. Afterwards, they were cooled to the investigated desorption temperature (40–70 °C) and, finally, exposed to a 30% CO_2_/N_2_ gas mixture (70 mL/min) for 90 min. CO_2_ adsorption capacity performance (mmol CO_2_/g ads) was determined from the weight gain of the investigated materials in the adsorption process.

## 4. Conclusions

In the current study, a KIT-6 mesoporous molecular sieve was successfully synthesized by using a triblock copolymer and a butanol mixture as reported in the literature. A KIT-6 Sil mesoporous material was prepared by grafting using APTES at three different concentrations: 20, 30 and 40 wt.% for surface functionalization with alkoxysilane-type molecules.

From the X-ray diffraction spectra, it was observed that the Ia3d cubic structure of a KIT-6 mesoporous molecular sieve was achieved. Adding APTES to the reaction mixture of the KIT-6 mesoporous molecular sieve caused a slight decrease in the tipping points to be observed, but the structure was still maintained for the KIT-6 Sil samples. The bands presented on the FT-IR spectra of KIT-6 at 3418 cm^−1^ and 680 cm^−1^ were identified as indicative of the presence of primary amines. The successful grafting of amines on the KIT-6 mesoporous molecular sieve was confirmed by the presence of N–H stretching and N–H bending vibrations.

Thermogravimetric analysis demonstrated the good thermal stability of the investigated KIT-6 even after functionalization with APTES (KIT-6 Sil 20, 30 and 40 wt.% samples). TPD measurements of the adsorption–desorption process of CO_2_ were performed between 40 and 70 °C, in agreement with the thermogravimetric analysis results.

CO_2_ adsorption isotherms of the functionalized KIT-6 Sil mesoporous material at 40–70 °C revealed that both the adsorption capacity (mmol CO_2_/g ads) and the efficiency of the amino groups (mol CO_2_/mol N_2_) increased with a decrease in the temperature. As can be seen from the adsorption–desorption cycle tests, KIT-6 Sil adsorbents show relatively good stability with lowered reduction in adsorption capacity. Therefore, after nine cycles of adsorption–desorption, mesoporous silica KIT-6 functionalized with APTES showed excellent regeneration and good thermal stability, which shows us that it can be considered as a potential adsorbent for CO_2_ adsorption.

## Figures and Tables

**Table 1:** The pore parameters of the amino-functionalized molecular sieves.

No.	Sample	Specific Surface Area (m^2^/g)	Pore Volume (mL/g)	Micropore Volume (ml/g)	Micropore Area (m^2^/g)	Pore Diameter BJH_Des_ (nm)
1	KIT—6	724.38	1.04	0.07	147.9	5.46
2	KIT—6 Sil 20%	372.0	0.49	0.048	74.2	4.94
3	KIT—6 Sil 30%	343.2	0.44	0.026	53.6	5.61
4	KIT—6 Sil 40%	265.6	0.34	0.003	8.62	4.91

**Table 2:** The amount of the captured CO_2_ at 40 °C using KIT-6 Sil 20%, KIT-6 Sil 30% and KIT-6 Sil 40%.

No.	Sample	Temperature (°C)	n_CO2_/g SiO_2_ (mmol/g SiO_2_)	n_CO2_/n_NH2_ (mmol/mmol)
1	KIT-6 Sil 20%	40	2.17	0.46
2	KIT-6 Sil 30%	40	2.23	0.512
3	KIT-6 Sil 40%	40	2.2	0.482

**Table 3:** Adsorption capacities for different functionalized mesoporous molecular sieves using different amine types.

Support	Amine Type	T(°C)/pCO_2_ (bar)	CO_2_ Adsorption (mmol/g Adsorbent)	References
MCM-41	MEA	25/1	0.89	Ahmed et al. [35]
MCM-41	DEA	25/1	0.80	Ahmed et al. [35]
MCM-41	TEA	25/1	0.63	Ahmed et al. [35]
MCM-41	APTES	30/1	1.20	Kassab et al. [36]
CARiACT	PEI	40	2.55	Gray et al. [37]
Diaion	PEI	40	2.40	Gray et al. [37]
PCSK-2-3-850	S heteroatoms	25/1	2.79	Guo et al. [38]
PCSK-2-3-850	S heteroatoms	25/0.15	0.79	Guo et al. [38]
SBA-15	PEHA	105/1	4.0	Kishor et al. [17]
KIT-6	PEHA	105/l	4.48	Kishor et al. [17]
HVMCM-41	PEHA	105/l	4.07	Kishor et al. [17]
KIT-6 Sil	APTES	40/1	2.23	This work

## Data Availability

The original contributions presented in the study are included in the article, further inquiries can be directed to the corresponding author.
